# Colonization or coinfection? A case report of *Mycobacterium avium* complex detected in a patient with pulmonary tuberculosis

**DOI:** 10.1128/asmcr.00081-24

**Published:** 2025-02-13

**Authors:** Tina I. Bui, Jackson Rowe, Allison R. Eberly

**Affiliations:** 1 Department of Pathology and Immunology, Washington University School of Medicine, St. Louis, Missouri, USA; Vanderbilt University Medical Center, Nashville, Tennessee, USA

**Keywords:** nontuberculous mycobacteria, tuberculosis, *Mycobacterium avium *complex

## Abstract

**Background:**

Nontuberculous mycobacteria (NTM) infections have been increasingly reported in the literature; however, isolation of both NTM and *Mycobacterium tuberculosis* complex (MTBC) in respiratory samples may indicate transient NTM colonization rather than true coinfection. Distinguishing between NTM colonization and true infection is necessary for guiding appropriate antimicrobial treatment regimen.

**Case Summary:**

Here, we describe a case of a 41-year-old immunocompetent female with pulmonary tuberculosis in which *Mycobacterium avium* and *Mycobacterium intracellulare*, both members of the *M. avium* complex (MAC), were independently identified with MTBC in two of three sputum cultures using Bruker Biotyper matrix-assisted laser desorption/ionization time-of-flight mass spectrometry (MALDI-TOF MS). After completing MTBC-targeted therapy, the patient fully returned to her baseline physical activity, and all subsequent cultures were negative for MTBC and MAC.

**Conclusion:**

This case study underscores the challenges in the adjudication of colonization from coinfection in mixed mycobacterial specimens. Although there are microbiological guidelines for diagnosing independent NTM infections and MTBC infections, there is an unmet need for guidance on diagnosing and treating NTM in patients with pulmonary tuberculosis.

## INTRODUCTION

Diagnosis of *Mycobacterium tuberculosis* complex (MTBC) and nontuberculous mycobacteria (NTM) coinfections is difficult due to their overlapping radiological and clinical presentations. Pulmonary coinfections with NTM and MTBC are rare but are increasingly documented in both immunocompromised and healthy individuals (1–12). Pulmonary disease attributable to NTM is on the rise globally (13), yet the rate of NTM and MTBC coinfections has only been described in MTBC-endemic regions (10, 14). In Taiwan, a study utilizing International Classification of Diseases codes estimated the rate of NTM-MTBC coinfections to be 2.8% between 2005 and 2013 (10), while in South Korea, 7% of tuberculosis (TB) patients had positive cultures for NTMs between 2003 and 2005 (14).

However, codetection of NTM and MTBC from respiratory specimens does not always indicate true NTM infection, as NTM is ubiquitous in the environment. Despite culture being the gold standard for the isolation of mycobacteria from respiratory cultures, some studies have used multiplex PCR for mycobacteria to detect both NTMs and MTBC and designated codetections as coinfections (7, 11). Other studies have reported NTM and MTBC coinfections based on single positive sputum cultures for NTM (14), which contradicts clinical practice guidelines published in 2020 by the American Thoracic Society (ATS), European Respiratory Society (ERS), European Society of Clinical Microbiology and Infectious Diseases (ESCMID), and Infectious Diseases Society of America (IDSA) (15). Discriminating between NTM colonization vs infection in concurrent with MTBC infection is important for patient treatment, as treatment regimens for NTM and MTBC are not analogous.

In this case report, we describe challenges in determining colonization vs coinfection when NTMs are isolated concurrently with MTBC from an immunocompetent individual. We also discuss the limitations of current recommendations for the diagnosis and treatment of NTM and MTBC coinfections.

## CASE PRESENTATION

A 41-year-old female presented to an outpatient clinic for evaluation of a chronic productive cough beginning over 3 years ago and a recent positive Interferon-Gamma Release Assay T-SPOT.TB test for TB. Subsequent chest radiograph showed diffuse bronchial and interstitial wall thickening throughout both lungs. Having migrated from the Philippines to the United States 15 years earlier, the patient recalled a negative screening chest radiograph upon immigration but later had a positive tuberculin skin test during pregnancy in the US. The follow-up chest radiograph at the time showed left apex pleural thickening but was negative for any features concerning TB, so treatment for latent TB infection was not initiated, and direct specimen nucleic acid amplification testing for detection of MTBC was not ordered.

**Fig 1:**
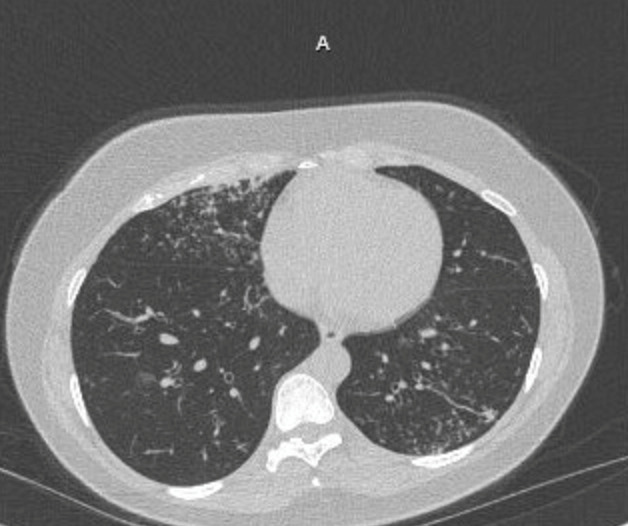
High-resolution CT scan of the chest. Extensive tree-in-bud nodules were seen throughout the bilateral lungs consistent with infectious etiology.

On presentation, the patient reported no other symptoms besides the chronic productive cough. Of note, the patient stated that a family member in the Philippines had recently been diagnosed with pulmonary TB, though the patient had no recent contact with them due to travel restrictions. The patient denied any additional risk factors for TB. Initial lab tests showed a normal white blood cell count and nonreactive fourth-generation HIV test. Serologic tests for *Histoplasma*, *Blastomyces*, and *Coccidioides* were all negative. Additional chest computed tomography (CT) showed extensive tree-in-bud nodularity with areas of consolidation in the lung bases and right apex concerning atypical mycobacterial infection or tuberculosis (Fig. 1).

**Fig 2:**
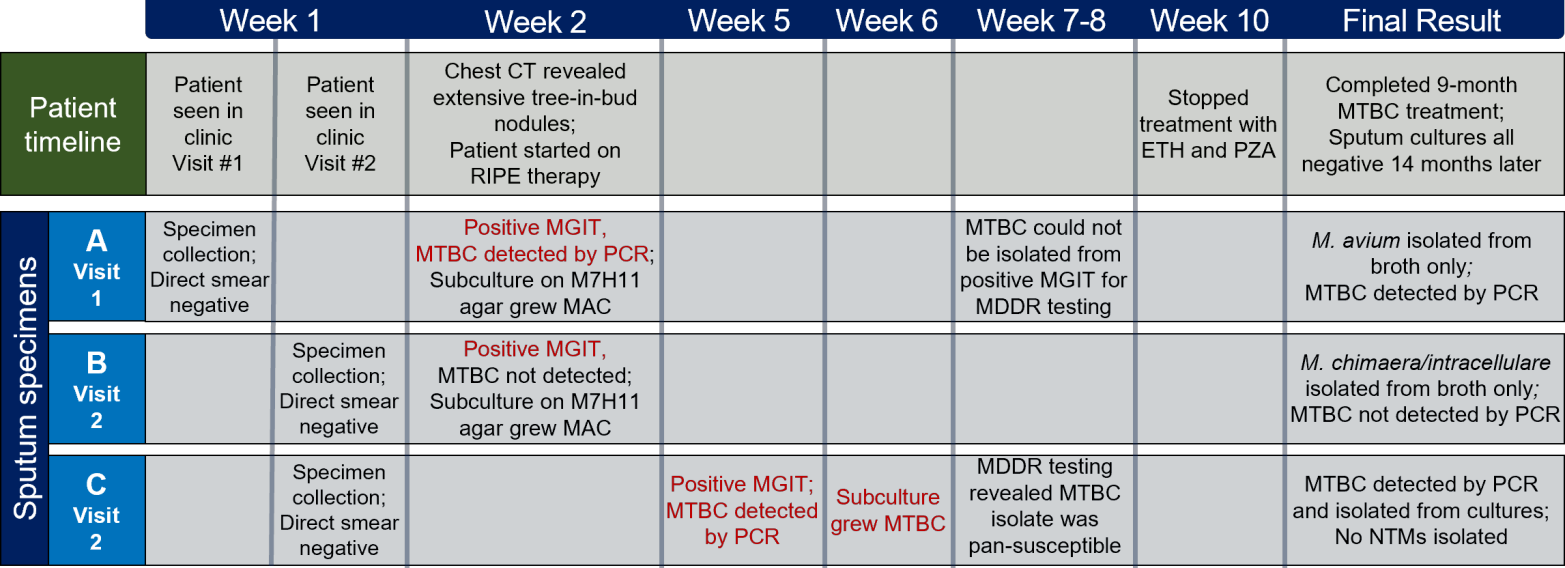
Timeline of patient care and microbiological testing. Sputum specimens were collected at two separate in-office visits, both in the late morning. Xpert MTB/RIF PCR was used to detect MTBC in positive MGITs. All growth on M7H11 was identified using Bruker Biotyper MALDI-TOF MS. MDDR testing was conducted by the Missouri State Public Health Laboratory. ETH, ethambutol; PZA, pyrazinamide.

Three sputum samples were collected at two separate clinic visits over a 4-day period in the late morning, all of which were sent for mycobacteria culture and staining. Figure 2 illustrates the timeline of culture results and corresponding critical patient care events. Acid-fast bacilli (AFB) were absent in all three specimens upon direct specimen fluorescent staining using auramine-rhodamine. The first liquid culture in the mycobacteria growth indicator tube (MGIT) flagged positive after 11 days of incubation, revealing abundant AFB by Kinyoun staining. At Barnes Jewish Hospital, the Xpert MTB/RIF PCR has been validated to test positive MGITs (no additional protocol modifications), which detected MTBC in our patient sample and led to the initiation of multi-drug therapy targeting MTBC with first-line antibiotics rifampin, isoniazid, pyrazinamide, and ethambutol (RIPE). However, molecular detection of drug resistance (MDDR) testing for MTBC was not conducted due to inability to isolate MTBC on subculture. Additionally, susceptibility testing on *Mycobacterium avium* complex (MAC) was not performed, although available upon clinician request. Following subculture onto Middlebrook 7H11 (M7H11) agar from the first positive MGIT, colonies were identified as *M. avium* using MALDI-TOF-MS (Bruker Biotyper). After 10 days of incubation, the second MGIT flagged positive and was AFB positive upon Kinyoun staining. MTBC was not detected by PCR in this MGIT. Colonies observed on subculture 3 days later were identified as *Mycobacterium chimaera/intracellulare* (subspecies not differentiated by Bruker Biotyper MALDI-TOF-MS, *Mycobacteria* Library RUO version 6). The clinical team did not request susceptibility testing on *M. chimaera/intracellulare*.

The final MGIT flagged positive after 4 weeks of incubation, with MTBC identified by MTB/RIF PCR and isolated from a subculture of the positive MGIT. MDDR testing confirmed that the MTBC isolate was pan-susceptible. Consequently, the patient continued therapy with only rifampin and isoniazid for an extended duration of 9 months, given the findings on chest CT. Following treatment completion, the patient reported symptom resolution and the ability to resume a prior level of physical activity. Multiple sputum samples collected 5 months post-treatment initiation were all negative.

## DISCUSSION

The isolation of both MTBC and NTM in respiratory samples, as illustrated in this case, presents a diagnostic and therapeutic challenge. NTM are ubiquitous environmental microorganisms found in soil and water. While pulmonary NTM infections are increasingly documented, particularly in immunocompromised patients, codetection of NTM in MTBC-positive respiratory specimens from immunocompetent patients can complicate clinical decision-making, as it may reflect transient colonization rather than true infection. In the case of this 41-year-old immunocompetent patient, two NTM species of MAC, *M. avium* and *M. intracellulare*, were recovered from separate sputum cultures that also tested positive for MTBC by Xpert MTB/RIF PCR. Although the MTBC result from PCR of the first positive MGIT could raise concerns since it was not isolated in culture, MTBC was detected across all five probes with low cycle threshold values ranging from 26 to 29, supporting the diagnosis of active MTBC infection.

MAC species are well-known pathogens responsible for pulmonary disease, and distinguishing between colonization and infection in patients already diagnosed with MTBC is essential. This distinction matters, as misinterpreting NTM colonization as an active infection can lead to a different antimicrobial treatment regimen. While a single culture-positive sample for MTBC is diagnostic for tuberculosis in a symptomatic patient, the same cannot be said for NTMs in respiratory specimens, as they are opportunistic pathogens. According to the ATS/ERS/ESCMID/IDSA guidelines published in 2020, the microbiological criteria for diagnosing pulmonary NTM require either positive cultures from ≥2 sputum specimens of the same NTM species (or subspecies in the case of *Mycobacterium abscessus*), positive culture from ≥1 bronchial wash or lavage specimen, or positive culture with transbronchial or lung biopsy specimen with supporting evidence of mycobacterial histopathology (15). The patient’s successful return to her baseline functional status following MTBC-targeted therapy supports two potential scenarios: (i) the NTM isolates in this case were more likely transient colonizers than contributors to the clinical disease, or (ii) the NTM isolates were susceptible to MTBC-targeted therapy. The former aligns with current guidelines, which emphasize the need for isolation of the same species across multiple positive sputum cultures to diagnose NTM pulmonary infections.

NTM and MTBC coinfections, though rare, can significantly impact laboratory testing, antimicrobial regimens, and patient outcomes. The implementation of MALDI-TOF MS has been instrumental in the rapid identification of bacteria. However, MALDI-TOF MS identification of NTM is more nuanced due to the high genetic similarity between NTM species. Over 200 NTM species have been characterized, and species-level identification is necessary to meet the microbiological criteria for diagnosing NTM pulmonary infection using sputum samples (15). NTM identification by Bruker Biotyper can vary between slowly growing and rapidly growing NTMs, where the accuracy for slowly growing NTMs was 87% compared to 99% for rapidly growing NTMs (16). Our patient had two sputum cultures that detected slowly growing NTMs with high confidence scores, suggesting that these isolates were of colonization rather than true infection based on diagnostic criteria set by the ATS/ERS/ESCMID/IDSA (15). However, the criteria for clinically relevant NTMs during pulmonary infections is not well defined due to the potential transient colonization. There is currently no defined culture or molecular threshold to discriminate between NTM colonization vs infection. The guidelines also recommend that molecular methods, such as isolate sequencing, should be used to determine species-level identification of clinically significant NTMs (15). Useful sequencing targets for mycobacteria identification include 16S, *rpoB*, and *hsp65*, although 16S rRNA gene sequencing may only resolve to the complex level, depending on the primers used (17–20). Sequencing was not performed on the two MAC isolates as molecular methods for identifying NTMs are expensive and may not be readily available, often requiring send-outs to reference laboratories.

Mixed cultures with NTM and MTBC can also influence rifampin resistance results during molecular testing of positive liquid cultures. In a previous case report, the coexistence of rifampin-resistant MAC and pan-susceptible MTBC resulted in false positive detection of rifampin resistance by Xpert MTB/RIF PCR (3). Consequently, treatment with RIPE was ineffective against MAC, leading to persistent pulmonary necrotizing inflammation. MAC infections are typically treated with a three-drug regimen consisting of a macrolide, a rifamycin, and ethambutol (15). Treatment for MTBC typically lasts for 6 months, whereas therapy for pulmonary MAC and other NTM infections is at least 12 months (15). Although MTBC therapy takes priority in the presence of codetection of NTM, NTM can cause chronic pulmonary disease if left untreated. While most patients with MTBC and MAC coinfections respond to MTBC treatment alone (2), as in our patient, additional therapy for MAC may be necessary if symptoms persist or in cases of disseminated disease. In some cases, isolation of NTMs can occur during MTBC-targeted therapy, suggesting that MTBC treatment alone may not be effective against NTM colonization (14). In summary, the latest guidelines have defined recommendations for NTM diagnosis and treatment but do not address NTM isolation during coinfections with MTBC (15). Thus, there is an unmet need for guidance on discriminating NTM colonization from coinfection when isolated concurrently with MTBC.

### Conclusion

In summary, this case illustrates the challenges associated with adjudicating NTM colonization from infection in patients with pulmonary tuberculosis. Additional studies are needed to assess codetection rates of NTM and MTBC from respiratory cultures in nonendemic areas to determine colonization rates. If NTMs are left untreated, TB patients may be at risk for chronic pulmonary disease. Therefore, further studies and guidance are needed to define NTM colonization versus coinfection and whether treatment specific for NTM is needed during coinfections with MTBC.
